# Preparation of Zinc Oxide-Starch Nanocomposite and Its Application on Coating

**DOI:** 10.1186/s11671-016-1404-y

**Published:** 2016-04-14

**Authors:** Jinxia Ma, Wenhua Zhu, Yajun Tian, Zhiguo Wang

**Affiliations:** Jiangsu Provincial Key Lab of Pulp and Paper Science and Technology, Nanjing Forestry University, 159 Longpan Road, Nanjing, 210037 China

**Keywords:** Nano-ZnO, ZnO-starch nanocomposite, Starch, Zinc chloride

## Abstract

A new production method of zinc oxide (ZnO)-starch nanocomposite was invented in this study. Starch was dissolved in zinc chloride (ZnCl_2_) solution (65 wt%) at 80 °C. Then, ZnO-starch nanocomposite was achieved when the pH of the solution was adjusted to 8.4 by NaOH solution (15 wt%). ZnO nanoparticles were also obtained when the generated ZnO-starch nanocomposite was calcined at 575 °C. The properties of ZnO-starch nanocomposite and ZnO nanoparticle were characterized by X-ray diffraction (XRD), scanning electron microscopy (SEM), and transmission electron microscopy (TEM). The results indicated that the sizes of ZnO-starch composite and ZnO particle were 40–60 nm. UV blocking effect was observed from both ZnO-starch nanocomposite and ZnO nanoparticle. The ZnO-starch nanocomposite was used to directly coat the surface of plain paper with a laboratory paper coater. The surface strength and smoothness of paper were improved by the coating of ZnO-starch nanocomposite. The antibacterial property was also identified from the coated paper.

## Background

Microbial contamination is a serious health issue in the food industries and hospital and medical settings. The development of agents or surface coatings with antimicrobial activity has gained increasing interest in recent years. Compared with organic antimicrobial agents such as quaternary ammonium salt and chlorine disinfectant, inorganic oxides have the advantages of robustness, long shelf life, high stability at higher temperatures or pressures, and the ability to withstand harsh processes. This has attracted much more focus on developing alternative inorganic oxides to substitute for the conventional organic compounds. Coatings are expected to be both stable and safe. Inorganic materials with antibacterial properties have been used as antimicrobial coatings on various devices to eliminate microorganisms on surfaces in the environment, community, and health care settings to help stop the spread of the diseases [1–4]. Among the inorganic oxides, zinc oxide (ZnO), titanium dioxide (TiO_2_), magnesium oxide (MgO), and calcium oxide (CaO) are not only stable under harsh conditions but also regarded as safe materials for humans and animals [3, 5–7]. Moreover, TiO_2_ and ZnO have been used in the formulations of various personal care products. Interestingly, ZnO shows antimicrobial activity without photoactivation, in contrast to TiO_2_, which requires photoactivation [5, 8–12]. Due to their small size (1–100 nm) and novel structures, ZnO nanoparticles exhibit significantly improved physical, chemical, and biological properties compared with bulk ZnO. ZnO nanoparticles are a multifunctional material with good catalytic, electrical, photochemical, and optical properties. They are suitable for a broad range of applications such as semiconductors, piezoelectric devices, field emission displays, gas sensors, biosensors, UV-shielding materials, photocatalytic degradation of pollutants, and antimicrobial treatments [1–4].

The methods for nanoscale ZnO production include vapor deposition, precipitation, the microemulsion method, the sol-gel process, and hydrothermal synthesis [1, 4, 7, 13]. The variety of methods enables obtaining particles with a variety of shapes, sizes, and spatial structures. Precipitation is a widely used method to obtain ZnO nanoparticles. The simplest route is an acid-base precipitation method for either coatings or biological applications. A zinc precursor solution such as zinc nitrate (Zn(NO_3_)_2_), zinc acetate (Zn(CH_3_COO)_2_), or zinc sulfate (ZnSO_4_) and an alkaline aqueous solution such as sodium hydroxide (NaOH), potassium hydroxide (KOH), or ammonium hydroxide (NH_4_OH) are prepared in deionized water. The acid solution is mixed with the base in varying proportions to achieve the desired molar hydrolysis ratio of Zn^2+^/OH^−^, and the precipitate is harvested, washed, and dried in a static air oven at 80–90 °C for several hours. Adding organic compounds such as polyvinyl alcohol (PVA) [14], polyethylene glycol (PEG) [15–17], starch [18, 19], sodium dodecyl sulfate (SDS), and cetyltrimethyl ammonium bromide (CTAB) [20] is increasingly common to control the growth of precipitated particles. These compounds affect not only nucleation and particle growth but also coagulation and flocculation of the particles. Therefore, the process of precipitation is controlled by parameters such as pH, temperature, precipitation time, and the addition of macromolecules. Wang et al. used aqueous solutions of ammonium bicarbonate (NH_4_HCO_3_) and zinc sulfate (ZnSO_4_·7H_2_O) to synthesize ZnO nanoparticles (*ca*. 40 nm) [21]. These authors also precipitated ZnO nanoparticles from zinc chloride (ZnCl_2_) and NH_4_OH in the presence of the cationic surfactant CTAB. The process was carried out at room temperature, and the resulting powder was calcined at 500 °C to remove residues of the surfactant. The product was small, well-dispersed 50-nm spherical nanoparticles composed of highly crystalline ZnO with a wurtzite structure [22]. Lanje et al. obtained ZnO nanoparticles from low-cost precursors such as ZnNO_3_ and NaOH [18]. To reduce agglomeration among the smaller particles, a starch was used, which contains many O-H functional groups and can bind to the surface of the nanoparticles at the initial nucleation stage.

ZnO nanoparticles can be used as part of organic/inorganic nanocomposite coatings in numerous industrial sectors, including food, textiles, packaging, environmental, health care, and medical care. ZnO nanoparticles have been incorporated with methyl cellulose (MC) [23], starch [24–26], acrylic binder [27], alginate [28], chitosan [29], and polyimide [30] to show strong antibacterial activity, dielectric properties, and UV absorbance. At present, there are some problems in the synthesis of ZnO nanoparticles and ZnO-starch composite materials such as the very low concentration of ZnO precursors. Most precursor concentrations are mmol/L (mM), and the highest concentration is 2 mol/L (M). Therefore, the process requires a large quantity of water or organic solvent. In addition, ZnO nanoparticles and ZnO-starch composites cannot be directly used as coatings but must be incorporated into polymer binders.

In this work, a ZnO-starch nanocomposite was synthesized using a facile process. The aqueous solution of 65 wt% zinc chloride (ZnCl_2_) is not only a precursor solution for ZnO nanoparticles but also a solvent for starch. The method involves first dissolving the starch by the ZnCl_2_ aqueous solution then adding NaOH. ZnCl_2_ is well known to be an effective swelling agent or solvent for cellulose [31–35]. To date, little information is available on ZnCl_2_ aqueous solutions as a starch solvent. Furthermore, few studies have focused on a route to synthesize a ZnO-starch nanocomposite with a ZnCl_2_ aqueous solution as both the precursor solution of ZnO nanoparticles and starch solvent. Moreover, the process to prepare the ZnO-starch nanocomposite requires less water. Therefore, this work aimed to develop a simple route to prepare a ZnO-starch nanocomposite and to characterize its morphology. The ZnO-starch nanocomposite was directly coated onto the surface of art base paper as a functional coating, and the antibacterial, surface strength, and optical properties of the coated paper were studied. In addition, the UV-vis absorbance and fluorescence behavior of the ZnO-starch nanocomposite and the ZnO nanoparticles were investigated to explore their potential applications in the field of UV-shielding materials.

## Methods

### Materials

All of the reagents are of analytical grade (i.e., purity higher than 99.9 %) and used without further purification. ZnCl_2_ and NaOH were analytical grade from Nanjing Chemical Reagent Factory, China. Starch and plain papers (art base papers) with base weight of 90 g/m^2^ were supplied by the Gold East Paper Company, China.

### Preparation of the ZnO-Starch Nanocomposite and ZnO Nanoparticle

First, 15 g of starch was completely dissolved in 100 g of 65 wt% ZnCl_2_ aqueous solution at 80 °C with 500 r/min constant stirring. Then, a 15 wt% NaOH aqueous solution was added drop-wise to the starch-zinc chloride aqueous solution with 500 r/min constant stirring to achieve a final pH value of 8.4. After the composite was aged for 30 min with constant stirring at 80 °C, the ZnO-starch nanocomposite was obtained. The ZnO nanoparticles were easily obtained by calcining the dried ZnO-starch nanocomposite at 575 °C for one hour.

### Characterization of the ZnO-Starch Nanocomposite and ZnO Nanoparticles

The X-ray diffraction patterns (XRD) were recorded on an X-ray diffractometer (Ultima IV, Japan) with nickel-filtered Cu K*α* (*λ* = 0.1542 nm) radiation. The diffracted intensities were recorded at 2*θ* angles from 10° to 70°. The crystallite grain size of the ZnO particles was estimated using the Scherrer equation, *D* = *kλ*/*β*cos*θ*, where *k* is a constant, generally considered 0.89 for ZnO, *λ* is the wavelength of the Cu K*α* radiation, 0.1542 nm, *β* is the full width at half maximum of the XRD peaks, and *θ* is the diffraction angle. The morphology of the ZnO-starch nanocomposite and ZnO nanoparticles was studied using a transmission electron microscope (TEM) (JEM-2100, JEOL, Japan) and a scanning electron microscope (SEM) (JSM-7600 F; JEOL, Tokyo, Japan). The UV blocking test of the ZnO-starch nanocomposite and ZnO nanoparticles was recorded with a UV-visible spectrophotometer (Lambda 950, Perkin Elmer, USA), and all scans were in the range from 200 to 800 nm. The fluorescence behavior of the ZnO-starch nanocomposite and ZnO nanoparticles was recorded with a spectrofluorimeter (LS55, Perkin Elmer, USA). The excitation maximum wavelength was 310 nm, and the emission scan was carried out in the range from 350 to 550 nm.

### Paper Coating by ZnO-Starch Nanocomposite

A K-type (K-303) coater was used to coat the ZnO-starch nanocomposite on art base papers. The concentration of the ZnO-starch composite was 17.2 wt%. The coating weight was controlled by adjusting the power and speed used in the coating process. All sheets were dried on a standard drier set to 105 °C and then stored in a conditioned environment (23 °C and 50 % relative humidity (RH)) for 48 hours until further analysis. The smoothness was measured in accordance with GB/T 456-2002 using a smoothness tester (DCP-BKP10K, Changjiang Papermaking Equipment Company, China). Brightness and gloss were measured in accordance with GB/T 7974-2002 and GB/T 8941-2007 using a brightness tester (YQ-Z-48A, Hangzhou Qingtong Instrument Company, China) and a gloss tester (WZL-300H, Hangzhou Qingtong Instrument Company, China), respectively. The IGT dry picking velocity was measured using AIC2-5 printability tester (Holland) with medium viscosity oil. To ensure the reliability of measurements, five parallel tests were performed for each sample.

### Antibacterial Assessment of Coated Papers

The inhibition effects of papers coated with the ZnO-starch nanocomposite were measured according to the GB/T 20944.1-2007 disk diffusion method. *Escherichia coli* (a type of gram-negative bacteria) and *Staphylococcus aureus* (a type of gram-positive bacteria) were used in the experimentation. The culture medium for the aforementioned microorganism was a mixture of 17 g of agar, 15 g of beef extracts, 5 g of peptone, and 5 g of NaCl in 1000 mL of water. The pH value was adjusted to 8.0 using 1 mol/L HCl or 1 mol/L NaOH. Then, 0.1 mL of the bacterial suspension (approximately 10^6^ CFU/mL) was spread on the agar plates, and circular samples of the coated papers (diameter 15 mm) were placed on the surface of agar. Then, the dishes were placed in an incubator at 37 °C for 24 h. The antibacterial activity was evaluated by measuring the diameter of the inhibition zones. Three replicates were carried out for each sample.

## Results and Discussion

### Preparation and Characterization of the ZnO-Starch Nanocomposite

**Fig. 1:**
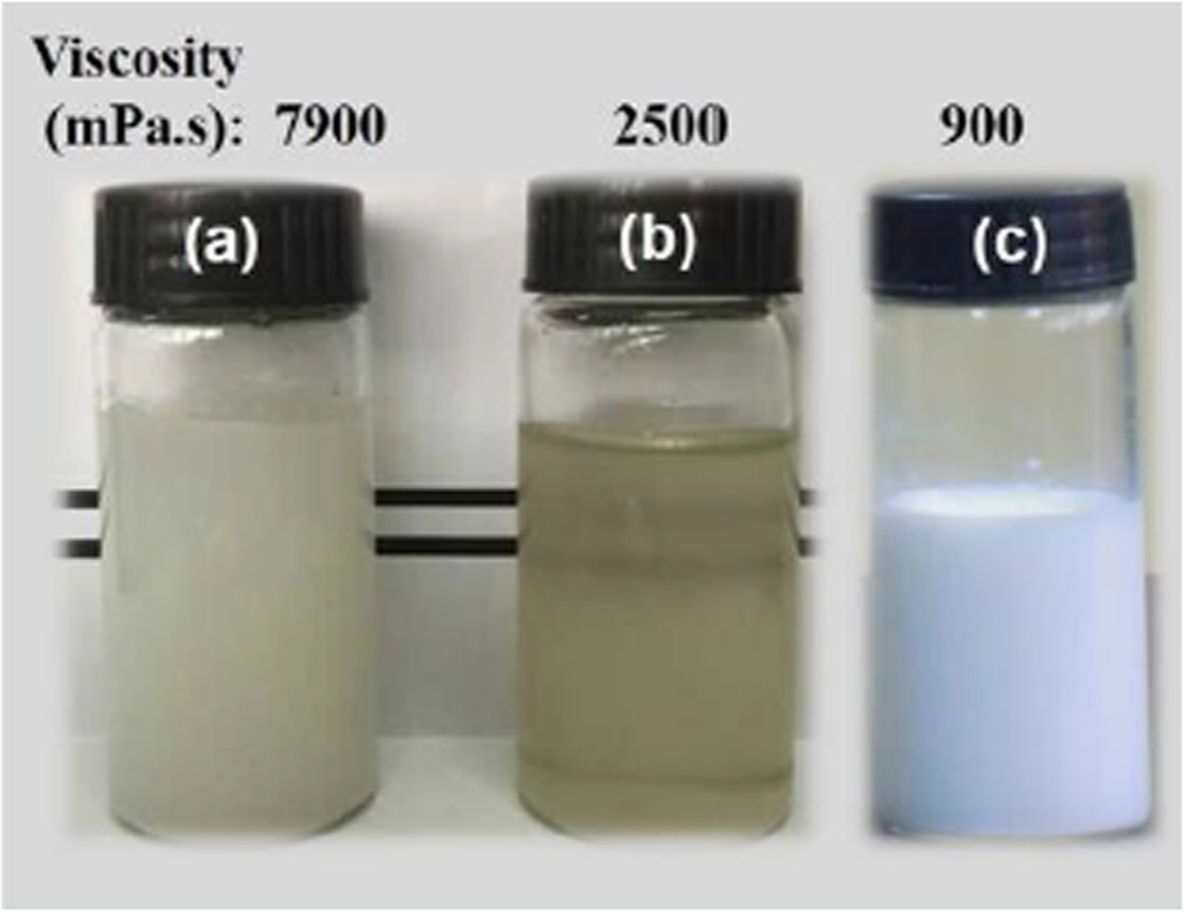
Photographs of samples **a** 8 wt% cooked starch solution, **b** starch-zinc chloride solution with 13 wt% concentration of starch, and **c**. ZnO-starch nanocomposite

**Fig. 2:**
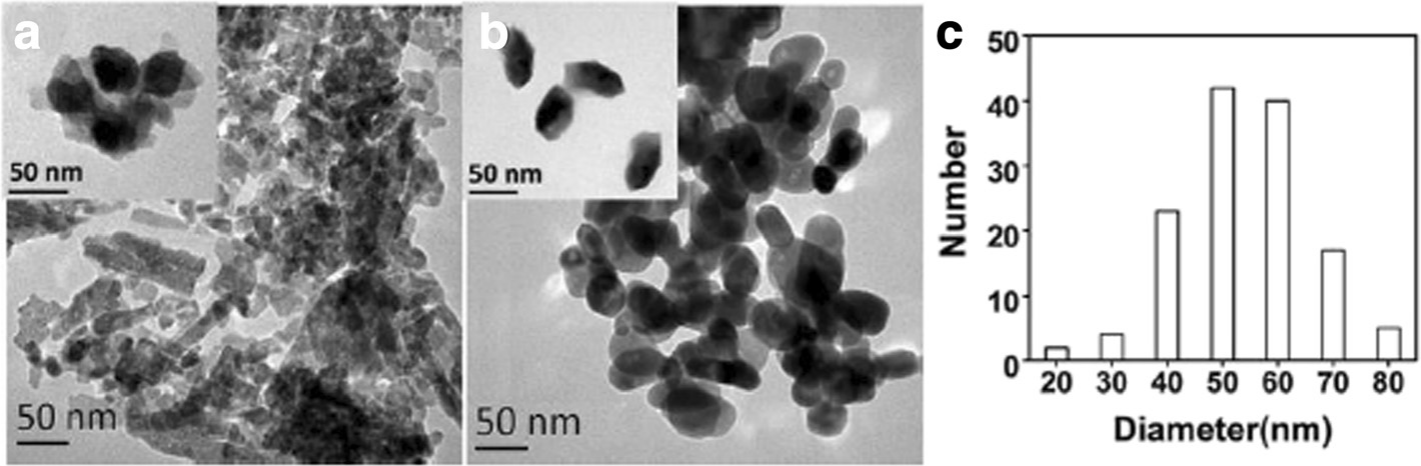
TEM images of **a** ZnO-starch nanocomposite, **b** ZnO nanoparticles, and **c** corresponding histogram of ZnO nanoparticles

As shown in Fig. 1, the starch-zinc chloride solution has higher transparency and lower viscosity compared with the cooked starch solution, even though the concentration of starch in the starch-zinc chloride solution (13 wt%) was higher than that of cooked starch solution (8 wt%). The results indicated that the starch was completely dissolved in 65 wt% ZnCl_2_ aqueous solution with a high degree of dissolution. Research on the dissolution mechanism of cellulose in the concentrated zinc chloride solution elucidated that zinc ions interact with the hydroxyl oxygen of the cellulose molecular chain to form a zinc-cellulose complex, which hydrolyzes the glycoside bonds [36, 37]. The crystalline intensity of starch is lower than that of cellulose. Zinc ions more easily interact with the hydroxyl oxygen of the starch molecular chain, which weakens the intermolecular hydrogen bonding, diminishes the crystalline regions of starch, and causes much more starch molecular chain dissolution, thereby resulting in a solution with lower viscosity. The ZnO-starch nanocomposite was prepared via the reaction between NaOH and the starch-zinc chloride solution. An aqueous solution of NaOH was added to the starch-zinc chloride solution to achieve a final pH value of 8.4, which led to the ZnO-starch nanocomposite after aging. The ZnO-starch nanocomposite appeared as an emulsion, similar to a traditional coating fluid as shown in Fig. 1c. The concentration of the starch dissolved in ZnCl_2_ solution was very high (13 wt%). The starch molecules in solution could interpenetrate to form nano-networks. In the initial nucleation stage, the O-H functional groups on the starch could bind to the surface of the ZnO particles. Meanwhile, the nano-networks formed by the starch molecules could control the ZnO particle growth, coagulation, and flocculation. The ZnO particles could not move freely, and they were dispersed uniformly in the nano-network. The particle size distribution of the ZnO-starch nanocomposite was uniform with diameters of approximately 35–45 nm as shown in Fig. 2a. Lanje et al. prepared ZnO nanoparticles using 0.1 M zinc nitrate and 0.2 M sodium hydroxide as precursors under 0.3 wt% cooked starch as a stabilizing agent. TEM images indicated that those ZnO nanoparticles were spherical in shape with an average size of 40 nm, and there was agglomeration of crystallites into ZnO nanoparticles [18]. The traditional route to synthesize ZnO nanoparticles needs a large amount of water due to the much lower concentration of precursors (mmol/L) [13–29]. Moreover, cooking starch needs very warm water. The concentration of the ZnO-starch composite prepared by our approach was 17.2 wt%. The present approach is simple and needs less water because of high concentration of starch and precursors.

### Characterizations and Properties of the ZnO-Starch Nanocomposite and ZnO Nanoparticle

**Fig. 3:**
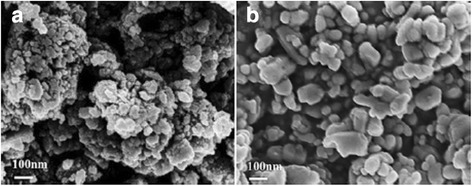
SEM images of **a** ZnO-starch nanocomposite and **b**. ZnO nanoparticles

The morphology of the ZnO-starch nanocomposite and ZnO particles was investigated with an electron microscope. Figure 2a illustrated that the ZnO-starch composite was not completely dispersed. It was possible due to high starch content in ZnO-starch composite. The inserted TEM image showed there was a layer of translucent material around the black crystalline particles. The ZnO particles could be bound with O-H groups on the starch molecules to form uniform ZnO-starch particles. The translucent material was possibly starch. The ZnO-starch particles were dispersed in the solution system at the nanoscale. ZnO-starch nanocomposite was uniform and the particle size approximately 35–45 nm. To obtain ZnO nanoparticles, the ZnO-starch nanocomposite was calcined at 575 °C. Figure 2b, c showed uniform and sporadic ZnO particles with approximately 50–60 nm in size. The agglomerated ZnO-starch nanocomposite and the dispersed ZnO nanoparticles are shown in the SEM images (Fig. 3). The particle sizes of the ZnO-starch nanocomposite and ZnO nanoparticles were approximately 50–150 nm, which were larger than the results from TEM images. The agglomeration of the ZnO-starch nanocomposite was possibly due to the presence of starch during the drying process. Figures 2 and 3 show that the particle shapes of the ZnO-starch nanocomposite and ZnO nanoparticles were only granular with no other shapes. Vigneshwaran et al. prepared zinc oxide-soluble starch nanocomposites using 0.1 M Zn(NO_3_)_2_ and 0.2 M NaOH as precursors under 0.5 wt% cooked starch as a stabilizing agent. In that study, SEM and TEM images demonstrated numerous ZnO nanoparticles ranging from 50 to 2000 nm in size embedded in soluble starch granules. Furthermore, there was agglomeration in the ZnO nanoparticles [19], which was possibly caused by the presence of starch. The ZnO-starch nanocomposite and ZnO nanoparticles prepared by our approach were also nanoscale and granular in shape. Therefore, the ZnO-starch nanocomposite and ZnO nanoparticles could be prepared under high concentrations of starch and precursors.

**Fig. 4:**
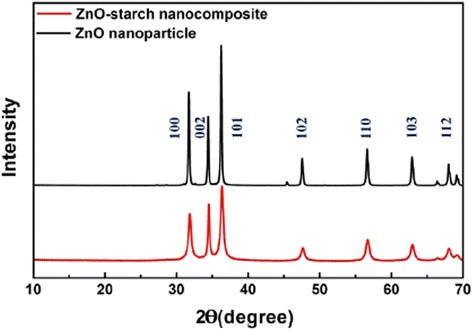
XRD patterns of ZnO-starch nanocomposite and ZnO nanoparticle

The XRD patterns of the ZnO-starch nanocomposite and ZnO nanoparticles are shown in Fig. 4. The peaks assigned to diffractions from various planes correspond to the wurtzite structure of ZnO. These results confirmed that ZnO was present in the ZnO-starch nanocomposite and ZnO nanoparticles. The observed broadening of the peaks for the ZnO-starch nanocomposite was mainly due to the nanoscale size effect. Calcination grew the ZnO nanoparticles to be larger than ZnO-starch nanocomposite. The ZnO-cooked starch nanocomposite and ZnO nanoparticles were prepared with Zn(NO_3_)_2_ and NaOH as precursors and cooked starch as a stabilizing agent. The reported X-ray diffraction patterns indicated not only diffraction peaks typical of the wurtzite ZnO crystal structure but also diffraction peaks of starch at 15° with low crystallinity [18, 19, 27]. The ZnO-starch nanocomposite and ZnO nanoparticles prepared by our approach had no low crystallinity diffraction peaks for starch, as shown in Fig. 4. The absence of the starch diffraction peaks because the starch completely dissolved into molecules. The crystallite sizes of ZnO nanoparticles were calculated from the Scherrer equation [18, 25]. The average particle size of ZnO-starch composite using the (100) and (101) reflections of ZnO was estimated to be 32 nm, while the average size of ZnO particles after the calcination of ZnO-starch nanocomposite was estimated to be 65 nm. These results agreed with the particle size obtained from the TEM images. The calcination caused an increase in the size of the ZnO particles.

**Fig. 5:**
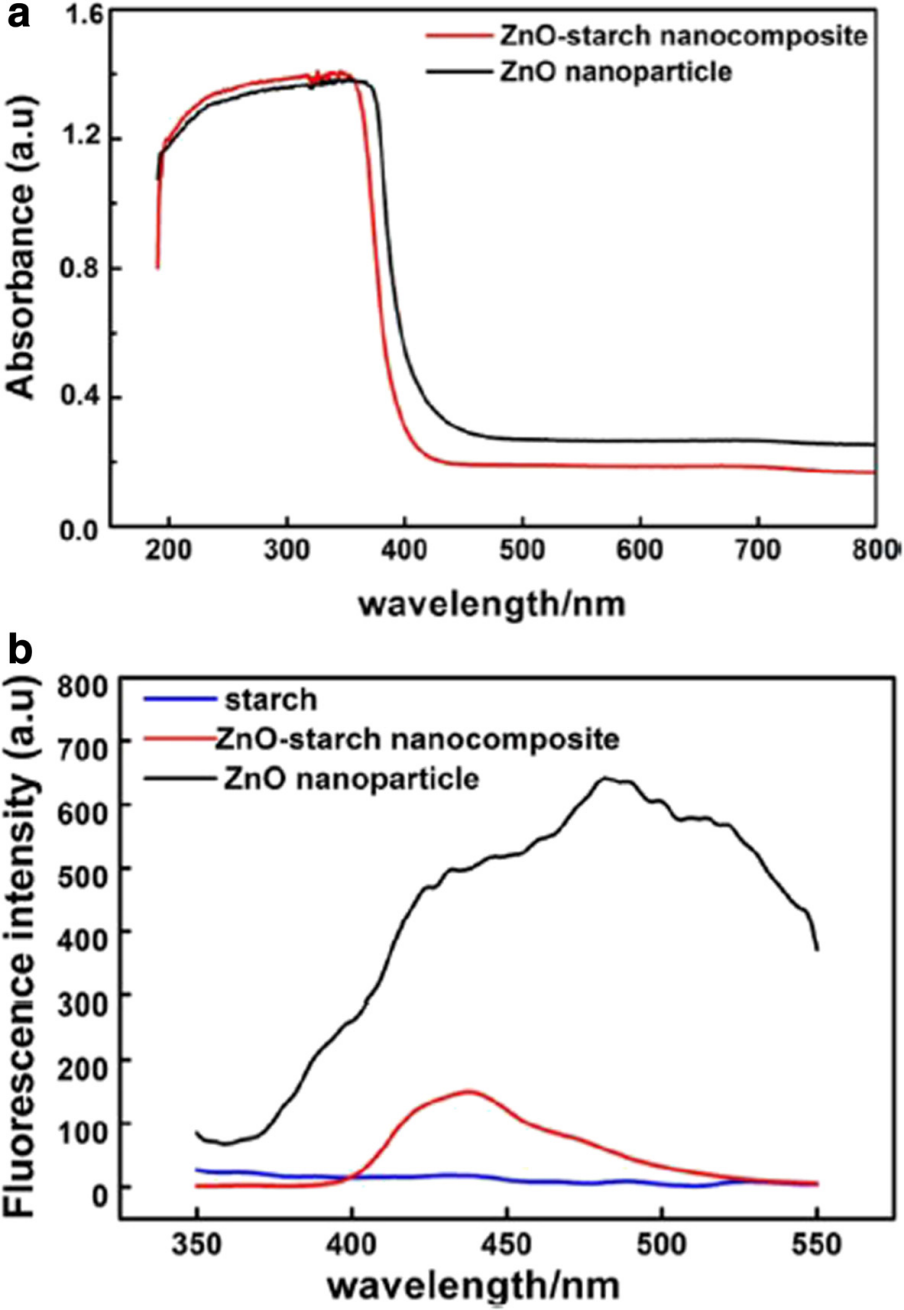
UV-vis absorption spectra (**a**) and fluorescence spectra (**b**) of ZnO-starch nanocomposite and ZnO nanoparticle. The excitation wavelength is 310 nm

ZnO nanoparticles are widely used as UV-shielding materials in many fields because of their excellent UV absorption property [25]. The UV-vis absorption spectra of the ZnO-starch nanocomposite and ZnO nanoparticles are shown in Fig. 5a. The ZnO-starch nanocomposite and ZnO nanoparticles exhibited strong absorption in the UV range (190–400 nm). Additionally, the absorption of ZnO nanoparticles had a broader spectrum range than that of the ZnO-starch nanocomposite. An absorption peak appeared at 350 nm for ZnO-starch nanocomposite, while the peak in the ZnO nanoparticles appeared at 380 nm. The shift in the absorption peak was possibly due to the presence of starch. To further investigate the UV-shielding property, the fluorescence behavior of the ZnO-starch nanocomposite and ZnO nanoparticle was investigated. As shown in Fig. 5b, the ZnO-starch nanocomposite and ZnO nanoparticles exhibited strong visible fluorescence at wavelengths of 440 and 485 nm, respectively, when excited by 310-nm ultraviolet radiations. Moreover, the luminescence emission of ZnO nanoparticles was markedly enhanced and broadened. To confirm that the visible fluorescence is from ZnO and not from the starch, the starch was also tested, which showed no emission peaks when excited by UV. Vigneshwaran et al. reported their ZnO-starch nanocomposite had a weak UV emission at 390 nm and three weak emitting bands (blue emission at 460 nm, a blue–green band at 490 nm and a green band at 530 nm) when excited by 325-nm ultraviolet radiations. They interpreted that the weak UV emission corresponds to the near band edge emission of the wide band gap of ZnO, due to the annihilation of excitons and a result of the quantum confinement effect. The green band emission corresponds to the singly ionized oxygen vacancy in ZnO and results from the recombination of a photo-generated hole with the single ionized charge state of this defect. The green emission also implies that there are few surface defects in the ZnO nanoparticles [19]. Prasad et al. prepared ZnO nanoparticles using 0.2 M zinc nitrate and 0.4 M sodium hydroxide as precursors under 0.5 wt% cooked starch as a stabilizing agent. ZnO nanoparticle was incorporated with cooked starch and coated on the plain paper. The papers coated with ZnO nanoparticles exhibited strong visible fluorescence at 440 nm wavelength when excited by UV. Similar fluorescence was observed for paper coated with bulk ZnO but with lesser intensity. They reported the emission band at 440 nm corresponds to band gap emission from zinc oxide. Our fluorescence spectra were similar with that of Prasad et al. [25]. The ZnO nanoparticles absorbed heavily in the UV region and its fluoresce with higher intensity in the visible region than ZnO-starch nanocomposite. This result may be ascribable to the fact that the calcination at 575 °C led to ZnO nanoparticles with a good crystallinity and less vacancies on their surface [38]. This property of UV absorption and simultaneous emission in the visible region helps to protect materials from UV exposure. This result demonstrates that the ZnO-starch nanocomposite and ZnO nanoparticles fabricated by this approach may effectively protect against UV light and could potentially be applied in UV-shielding materials.

### Effect of Coating on Paper Properties

The ZnO-cooked starch nanocomposite and ZnO nanoparticles prepared via the acid-base precipitation method could not be used directly as a coating. They had to be incorporated with polymer binders [18, 19, 23–30]. The ZnO-starch nanocomposite synthesized by the present approach could be directly used as an organic/inorganic nanocomposite coating, and it was coated onto the surface of plain paper. The optical and surface characteristics of the coated papers are given in Table 1.

**Table 1:** Effect of coating on paper properties

Types of coating	Coating weight (g/m^2^)	Brightness (%)	Gloss (%)	Smoothness (s)	Picking velocity (cm/s)
Base paper	0	97.80	3.5	1.7	137
Cooked starch	2.52	96.98	2.8	1.9	182
ZnO nanoparticle mixture with cooked starch	2.38	96.73	2.5	1.9	218
ZnO-starch nanocomposite	2.13	96.90	2.1	2.1	254

Table 1 indicates that the coating made the paper brightness and gloss decrease, while the picking velocity and smoothness increased. The presence of starch on the paper surface or in the art base paper affected the brightness and gloss due to an increase in the paper transparency. Moreover, ZnO nanoparticles could reduce the reflectance of incident light on the papers. The increased smoothness and picking velocity were also caused by the starch and ZnO nanoparticles. The highest smoothness and picking velocity was achieved by the paper coated with the ZnO-starch nanocomposite, while the ZnO nanoparticle mixture with cooked starch had lower smoothness and picking velocity than the ZnO-starch nanocomposite. This revealed that the ZnO nanoparticles had a high specific surface area and could not be completely and uniformly dispersed in the cooked starch. For the ZnO-starch nanocomposite, the ZnO nanoparticles were dispersed uniformly in the starch network, and the dissolved starch could uniformly bind with the ZnO nanoparticles and fibers in the paper. This binding resulted in the best surface strength and smoothness.

**Fig. 6:**
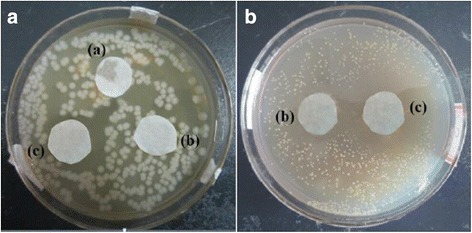
Antibacterial properties of papers (**A** against *E. coli*, **B** against *S. aureus*, (*a*) base paper, (*b*) paper coated with mixture of ZnO nanoparticle and cooked starch, and (*c*) paper coated with ZnO-starch nanocomposite)

Even in the absence of light, ZnO is an efficient antibacterial agent for a broad range of target bacteria [8, 9, 39]. The antibacterial activity of papers coated with the ZnO-starch nanocomposite was also studied. Figure 6 shows that there were apparent inhibition zones against *S. aureus* around the base paper, but there were not apparent inhibition zones around samples for *E. coli*. The inhibition zone against *E. coli* around the paper coated with the ZnO-starch nanocomposite was much larger than that of the paper coated with ZnO nanoparticle mixture with cooked starch. Ameer Azam et al. reported that gram-negative bacterial strains of *E. coli* had smaller inhibition zones than those of gram-positive bacterial strains *S. aureus* in the presence of ZnO nanoparticles [40]. This indicated that the *E. coli* strain exhibited a higher resistance to ZnO than the *S. aureus* strain. The present results are consistent with the results of Azam et al. In short, the ZnO-starch nanocomposite exhibited distinct antibacterial efficiency against *S. aureus*.

## Conclusions

The ZnO-starch nanocomposites were prepared by a facile process in which NaOH was added to a starch-zinc chloride solution at 80 °C to adjust the pH value to 8.4. The size of the ZnO-starch nanocomposites and ZnO nanoparticles was approximately 40–60 nm. The ZnO-starch nanocomposite and ZnO nanoparticles exhibited a UV blocking property, strong visible fluorescence and an efficient antibacterial capability. Moreover, The ZnO-starch nanocomposite could be directly coated onto the surface of plain paper. The picking velocity and smoothness of papers coated with the ZnO-starch nanocomposite were superior to those of papers coated by a mixture of ZnO nanoparticles with cooked starch and with cooked starch alone.
